# HTLV Tax repress nonsense mediated mRNA decay by inhibiting the ATPase activity of UPF1

**DOI:** 10.1186/1742-4690-12-S1-P36

**Published:** 2015-08-28

**Authors:** Mocquet Vincent, Fiorini Francesca, Robin Jean-Philippe, LeHir Hervé, Jalinot Pierre

**Affiliations:** 1 Laboratoire de Biologie Moléculaire de la Cellule, UMR 5239, CNRS, Ecole Normale Supérieure, 69364 Lyon Cedex 07, France

## 

The Nonsense Mediated mRNA Decay (NMD) is a mRNA quality surveillance pathway that regulates the stability of mRNAs harboring a premature termination codon, avoiding the production of truncated proteins with potential oncogenic properties. NMD regulates also the expression levels of 10 to 15% of human genes such as those regulating the hematopoietic cell differentiation, the maintenance of chromosome structure and function and retroviral RNA. Due to its role in the control of expression of gene involved in the immune response, and the targeting of viral RNA, NMD is now also considered as a viral restriction mechanism. Describing the details of the interplay between HTLV and the NMD is then important to better understand how the virus evades this surveillance and to determine a role of the NMD in the associated pathologies. At the center of the NMD pathway, UPF1 orchestrates a dynamic network of interactions leading to its own structural rearrangement as well as the ribonucleoprotein (RNP) remodeling. In particular, its N-terminal domain (CH domain) repress its ATPase and helicase acitvities held by the central domain (HD domain) until it interacts with the other core factor UPF2 in the early steps of NMD. We showed for the first time that the retrovirus HTLV-1 was able to protect its RNA and modulate some of its transcriptional activators by inhibiting NMD. Now we focuse our attention in deciphering the mechanistics of this inhibition. Thus we demonstrate that HTLV-1 Tax protein targets the RNA helicase UPF1. We found that in cells transfected with a complete provirus, Tax is associated to the NMD sensitive mRNA in an UPF1 dependent manner. This interaction between Tax and the HD domain of UPF1 inhibits the ATPase acivity of UPF1. The homology in amine acid sequence as well as competition assays lead us to the conclusion that Tax could mimics the CH domain in order to maintain a repressive effect even in the latter steps of the pathway. This conclusion is supported in vivo by the accumulation of intermediary complexes stalled on an NMD target. Finally we identified the Tax binding site necessary for this inhibition. Then those data provide structural insights on the regulation of the RNA helicase UPF1 functionning as well as a precise mechanistic of Tax inhibitory effect during NMD.

